# Biomarkers of Alzheimer’s disease modification using adaptive cognitive assessments to improve responsiveness—a simulation study

**DOI:** 10.3389/fnins.2025.1653261

**Published:** 2025-09-23

**Authors:** Antonio Rodríguez-Romero, Shibeshih Belachew, Emmanuel Bartholomé, Claudia Mazzà, Óscar Reyes, Carlos Luque, Silvan Pless, Corrado Bernasconi

**Affiliations:** ^1^Indivi AG, Basel, Switzerland; ^2^Research Center for Clinical Neuroimmunology and Neuroscience Basel (RC2NB), University Hospital Basel, University of Basel, Basel, Switzerland

**Keywords:** cognition, neurodegenerative diseases, adaptive test, biomarker, endpoint, simulation—computers, responsiveness, digital health

## Abstract

**Introduction:**

Clinical studies assessing cognition in Alzheimer’s and other neurodegenerative diseases require endpoints that are sensitive to treatment response across a broad range of cognitive abilities. However, responsiveness of conventional cognitive assessments typically varies with the performance level, especially due to non-linearities such as floor or ceiling effects. Here, we evaluate 6 newly developed smartphone-based and gamified Adaptive Cognitive Assessments (ACAs) entailing a system of dynamic difficulty adaptation to individual performance, which is expected to improve adherence but also measurement properties. Deployment of such ACAs to maximize their discriminative ability in comparative studies requires exploration of many free parameters and complex dynamics.

**Methods:**

In simulations of cohorts of patients with cognitive impairment, we compared two ACAs paradigms: after 14 daily tests allowing performance-based difficulty adaptation, the difficulty level was either (1) fixed or (2) kept adaptive for a period of 4 years with weekly testing. Sensitivity to between-group effects was assessed in cohorts characterized by cognitive decline observed in neurodegenerative diseases.

**Results:**

The discriminative ability of the two paradigms depends on features of the study design and subjects. At study end, the adaptive difficulty paradigm clearly outperformed the fixed-difficulty paradigm in terms of responsiveness for cognitive decline rates >2.5% per year.

**Discussion:**

ACA can increase biomarker responsiveness to treatment effects over fixed difficulty. ACA deployment should be guided by study and assessment features, including duration, expected cognitive decline rates and effect size. In the high-dimensional parameter space of ACA instruments, study simulations are indispensable to identify suitable deployment strategies.

## 1 Introduction

In the area of neurodegenerative diseases, recent years have seen a constant increase in the number of interventional clinical studies that target cognitive function as a key outcome of interest. Measures of cognition are among the main study endpoints in Alzheimer’s Disease (AD) (Mintun et al., 2021; van Dyck et al., 2023) and are becoming increasingly relevant also in other neurodegenerative conditions, such as Parkinson’s Disease (PD) with its variants (Aarsland et al., 2017), Multiple Sclerosis (MS) (Strober et al., 2019), Huntington’s Disease (HD) (Paulsen et al., 2013) as well as in psychiatric diseases. In clinical study cohorts, cognitive ability/performance and cognitive decline often vary over a broad range (Mintun et al., 2021; van Dyck et al., 2023; Butler et al., 2025). Large between-subject variability constitutes a serious challenge to the sensitivity of experiments because the responsiveness of conventional tools typically depends on the performance level as well as several other factors (Albert et al., 2011; Elkana et al., 2016). This is due to floor or ceiling effects (Butler et al., 2025) or other types of nonlinearities in the mapping of cognitive ability to test results. In addition, prolonged learning/practice effects are commonly observed, especially in subjects with normal or only slightly impaired cognitive ability, and often mask cognitive decline by yielding apparent improvement trajectories. For example, in MS, cognitive performance measured by the symbol-digit modalities test (SDMT) or the paced auditory serial addition test (PASAT) generally increases during the entire course of a typical interventional study (generally of about 2 years duration), even in control arms (Benedict et al., 2025; Leavitt et al., 2025).

Compared to standard in-clinic neuropsychological tests, the use of smartphone-based self-assessments at home may improve measurement reliability, ecological validity and accuracy, while enabling an increased assessment density, which may also result in a rapid saturation of any learning or practice effects (Pham et al., 2021; Lam et al., 2022). Gamification and adaptive features are expected to provide relevant advantages in terms of compliance in longitudinal studies. Importantly, adaptation may additionally confer beneficial psychometric properties to the tests, especially concerning longitudinal responsiveness and sensitivity to treatment effects. Nonetheless, this aspect appears to be neglected in the literature on adaptive cognitive tests, where development efforts typically focused on diagnostic tools that reduce the testing burden (Wouters et al., 2009; Gibbons et al., 2024) rather than tools designed to detect longitudinal change and treatment effects. Longitudinal responsiveness would be a necessary feature of instruments to be used as pharmacodynamic response biomarkers indicative of disease modification (Cummings et al., 2025).

In this work, we evaluate CoGames, a battery of 6 recently developed smartphone-based and gamified adaptive cognitive assessments (ACAs), which entail a system of dynamic difficulty adaptation to individual performance over repeated administrations (Pless et al., 2023, 2025). The ACA instruments assess working memory, information processing speed, short-term (visuo-spatial) memory, semantic recognition, executive functioning, cognitive flexibility, sustained and divided attention, and psychomotor speed.

In adaptive cognitive assessment paradigms, the change in assessment difficulty along with performance creates an entanglement between the performance metrics (scores) and the task difficulty level, which makes the definition of longitudinal measures of cognitive decline multidimensional by construction. In this sense, optimal deployment and endpoint definitions that maximize the responsiveness and discriminative ability of ACAs in clinical studies remain to be identified. Due to the highly dimensional parameter space, which experimental data and statistical models can only cover to a minimal extent, study simulations are an essential strategy to explore relevant practical scenarios as well as general properties of ACAs. This work hence describes a study simulation tool and investigates the instrument responsiveness resulting from different deployment strategies of ACAs, with a focus on long-term investigations of cognitive decline in Alzheimer’s disease.

## 2 Materials and methods

### 2.1 Simulation tool

Indivi’s CoGames battery (Supplementary Figure S1 and Supplementary Table S1) includes six distinct ACA instruments, in which subjects are invited to perform ACA runs iteratively according to a protocol-specific schedule. Based on performance (termed ‘score’), at each run (i.e., test execution) at a given difficulty level (termed rank), subjects are assigned a rank for the next run. The different instruments have between 5 and 8 ranks.

We developed a tool to stochastically simulate trajectories of individual subjects through a series of runs of each CoGames ACA instrument and implemented it in R (R Core Team, 2021), version 4.5.0. The tool generates individual trajectories (T), i.e., for each patient i and ACA instrument, a sequence of N runs:


$$T_{i,N}={\left(\mathrm{ran}k_{i,k},\text{scor}e_{i,k}\right)}_{k=1,\ldots ,N}$$

From an initial rank (that can be chosen freely), trajectories are calculated iteratively by generating (1) the current score, based on a probabilistic individual subject model, which for each test consists of the distribution of the individual subject’s score at any given run in a trajectory, and (2) the next rank, determined from a study model. This consists of a set of deterministic rank transition rules that identify the next rank as a function of the current test score and the trajectory up to that run.

#### 2.1.1 Individual subject model

Since the models are built in the same way for all 6 CoGames ACA instruments (with the exception of the short-term memory ACA «Treasure Hunt», as noted below), the approach will be described without reference to a specific test.

In the iterative simulation of individual subject trajectories through a set of runs, for a subject *i* who performs the *j*-th test in a trajectory and has been assigned $\mathrm{ran}k_{i,j}$ , the test score ( $\text{scor}e_{i,j}$ ) is drawn from a probability distribution (which we generically call *f*) that depends on the past runs in the trajectory:


$$\text{scor}e_{i,j}=f(\mathrm{ran}k_{i,j},T_{i,j-1})$$

These distributions define individual subject models, which need to be constructed for each cohort to be simulated. An example is presented in Section 2.2.1, where the patient model is based on normal distributions. In general, the distributions *f*, are obtained by: (1) defining a (marginal) baseline distribution for each rank (typically from experimental data) used for the first test execution at each rank, (2) tuning the parameters of the marginal distribution for subsequent runs to model longitudinal performance changes, such as short-term learning/practice effects or steady cognitive decline, and (3) incorporating within-subject relationships (correlation) to model the stability of individual trajectories, so that the current score is drawn from a conditional distribution accounting for the past. Parameters of these distributions can be defined on the individual subject level. Short-term learning effects (including also practice effects) and steady cognitive decline were modeled over time by deterministic functions used as multipliers for the expectation of the score distribution at each run.

More specifically, short-term learning effects were incorporated using the following (half) sigmoidal function of the run (called *L*) with a value of 1 at the first run, an asymptote at max (reflecting 100 * (*max* − 1) % improvement) and a *rate* parameter determining how quickly the asymptote is approached.


$$L(\max,\text{rate},\mathrm{run})=\frac{\max}{1+\exp(-\text{rate}*(\mathrm{run}-1-\ln(\max-1)/\text{rate}))}$$

**Figure 1:**
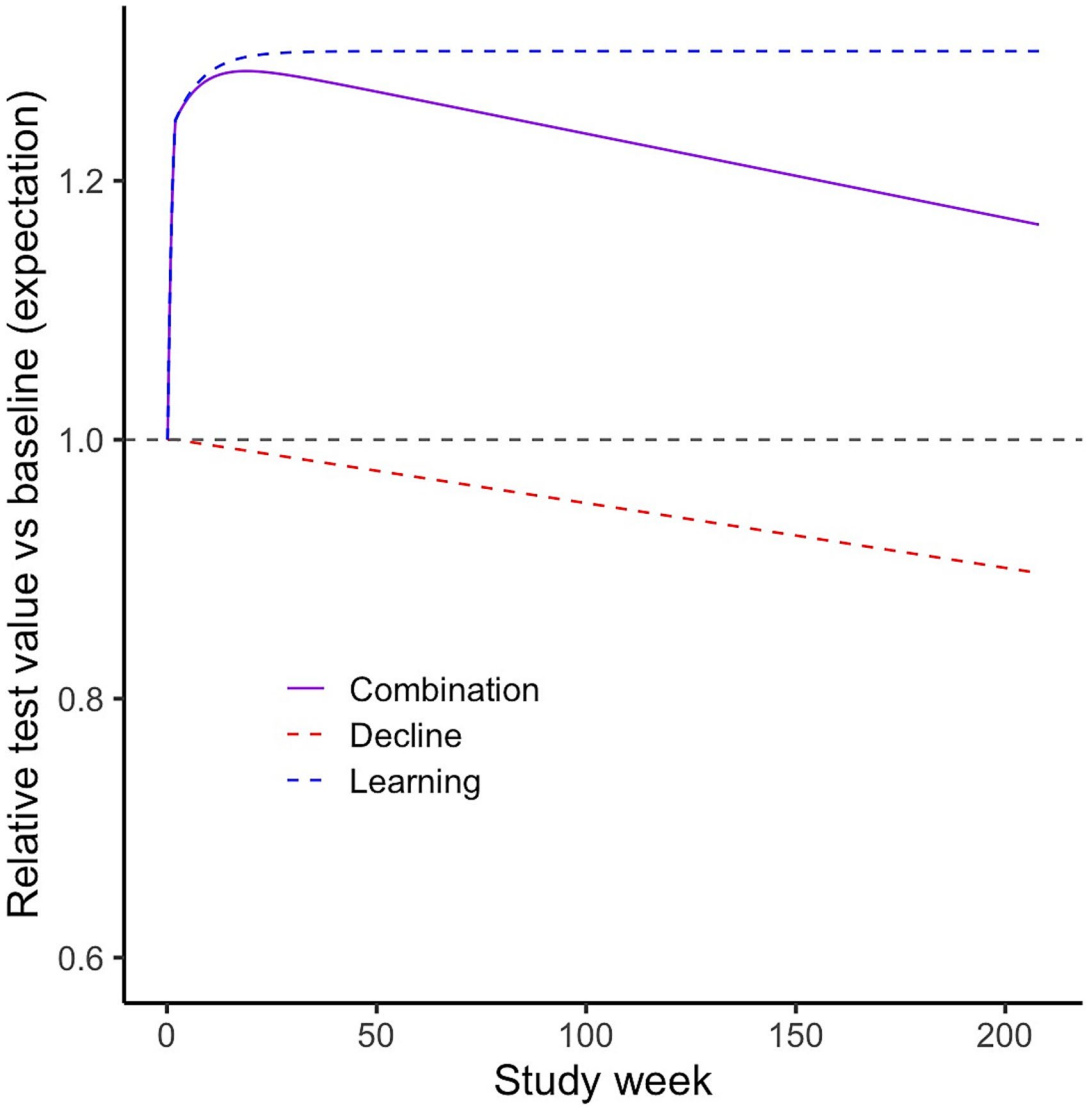
Learning, cognitive decline functions and their combined effect. Example based on the following parameters: Study: 14-day run-in, then weekly tests. Learning: max. 1.3, rate 0.15, weekly decline rate 0.0005 (i.e., modest decline of ~2.5%/year).

Cognitive decline was modeled for all tests and ranks as the constant 1 (no decline) for a certain initial number of runs and thereafter a linear function of the run (constant decline), until the minimum of 0 (not reached in the scenarios presented in this work). The initial period with stable cognition is used to represent studies including a run-in phase with intensive test administration (e.g., daily for 2 weeks) before subjects are moved to a regular schedule with more distanced assessments (e.g., weekly, monthly). For a regular testing schedule, which we assume for our typical simulated studies (after a short initial intensive run-in phase, see Section 2.2.2), a linear decline function reflects constant cognitive decline over time. See Figure 1 for an example of learning and decline functions and their combined effect.

#### 2.1.2 Study model

The study model (function sm ) describes the decision rules determining the rank assigned to a subject who has obtained a specific score at a given test run in a trajectory. For a subject *i* who has performed the *j*-th test in trajectory, i.e., who has been assigned to $\mathrm{ran}k_{i,j}$ and obtained $\text{scor}e_{i,j}$ the next rank described as a deterministic function of the trajectory


$$\mathrm{ran}k_{i,j+1}(\text{scor}e_{i,j})=\mathrm{sm}(T_{i,j})$$

In a typical implementation (see Section 2.2.2), this decision rule assigns a higher rank (if available for the specific instrument) to the subjects if the score has exceeded specific cutoffs for a given number of the latest runs in a row, or a lower rank if the score was repeatedly under some other cutoffs. Note that, in our parameterization, the time between administrations (e.g., weekly, monthly) is not explicitly mapped in the study model but would have to be incorporated indirectly by manipulating other parameters such as learning and decline functions or longitudinal correlations.

### 2.2 Simulations

#### 2.2.1 Parameters of the patient model

At any given run in a trajectory, a score is initially obtained from a (conditional) normal distribution. To reflect the range of possible values in the features being modeled, if the obtained score is <0, it is set to 0. An upper limit was only set for Treasure Hunt instrument, where the score is limited to 100, consistently with its specific derived candidate biomarker (relative correctness of the execution) being bound to 0–100%. While the lower limit does not have practical relevance in the original data and in the simulations, the upper limit in Treasure Hunt reflects a substantial ceiling effect, which in the data was present especially in the lower ranks, where the test is rather easy for the subjects with unimpaired cognitive abilities.

The parameters of the baseline distributions were supposed to reflect a cohort with a rather broad range of cognitive capacity and, on average, with mild cognitive impairment. As a data source for patients in whom cognition is expected to be slightly impaired (Benedict et al., 2025; Nasirzadeh et al., 2024), we used patients with Multiple Sclerosis at an overall low level of disability from an observational study (NCT05009160) (Research Center for Clinical Neuroimmunology and Neuroscience Basel, 2025).

As in the referenced study some of the upper ranks were disabled for the ACA, the parameters of the distribution for the missing ranks were obtained by extrapolation using data from healthy volunteers (HVs) (Pless et al., 2025) in the following way: for each game, for all the ranks that were in common, we first computed the mean (over ranks) of the percentage difference in rank-specific sample means between the patients and the HVs. This percentage (reflecting a lower performance in patients vs. HV) was applied to the mean in HV to derive the mean for ranks where no patient data were available. Similarly, for the standard deviation, the extrapolation from HV to patients was done by computing the mean coefficient of variation in patients, which was multiplied by the mean in HVs. This was justified by the observation of a quite stable coefficient of variation across the different situations. Notably, the distributions were computed using the first test execution at each rank in these studies. From the second run onwards at a given rank, the parameters were taken from the second test execution in the reference study, as there was generally a slight change from the first to the second execution. The parameters of the patient model can be found in the Supplementary material. The studies involving humans were approved by Ethikkommission Nordwest- und Zentralschweiz (EKNZ), Basel, Switzerland (BASEC ID 2021 D0040). The studies were conducted in accordance with the local legislation and institutional requirements. The participants provided their written informed consent to participate in the studies.

The learning function had maximum = 1.3 and rate = 0.2 as parameters, corresponding to an improvement from baseline approaching 30% with 80% of the effect reached after 14 runs (simulating a run-in baselining phase of 2 weeks of daily runs). These may be conservative estimates compared to data from the literature, considering the dense initial testing schedule in a population of elderly subjects with modest cognitive impairment (Bartels et al., 2010; Zheng et al., 2022). Cognitive decline was modeled for all tests as the constant 1 (no decline) until the 14th run and a linear function of the run thereafter, with rates that were varied in different simulations.

In the iterative simulation of trajectories, when the rank changed, the new score was obtained from the marginal normal distribution appropriate for the run (i.e., accounting for learning and decline effects). In subsequent runs at the same rank, the new score accounted for the previous score and the within-subject correlation. For each game and rank, this correlation was estimated from the patients or the HV data (where no patient data were available) using the first two available test runs. Correlations were generally high, implying a good stability of trajectories compared to the overall variability in the cohort: the mean over all games and ranks was 0.76, and <10% of the values were under 0.5 (mostly from the lowest ranks of Treasure Hunt, where the mentioned non-linear effects appear).

A new score (at run *j +* 1) was computed conditionally on the observation of the previous score (run *j*) by assuming bivariate normal distribution for the two scores, with their rank and run-specific means (*μ*) and standard deviations (*σ*) and the rank-specific correlation (𝜌): $N(\mu_{j},\sigma_{j}^{2},\mu_{j+1},\sigma_{j+1}^{2},\rho ).$ Parameters of the univariate normal distribution of the new score score_*j*+1_ conditional on the observation of the previous one ( ${\text{score}}_{j}$ ) were then:


$$\text{Mean}=\mu_{j+1}+\frac{\rho \;\sigma_{j+1}(\;{\text{score}}_{j}-\mu_{j})}{\;\sigma_{j}};\text{Variance}=(1-\rho^{2})\sigma_{j+1}^{2}$$

In practice, from the second run at each rank, the time series was generated by a non-stationary (due to the learning and decline trends), first-order autoregressive process.

#### 2.2.2 Parameters of the study model

Two study types with different administration paradigms of smartphone-based ACAs were modeled and compared. In a first common run-in phase of the study, for the first 14 runs (reflecting daily testing for 2 weeks), subject-level performance-based difficulty adaptation was allowed at every run, i.e., if the specific cutoff for rank change was reached at the specific run. Then in a second phase of the study (the evaluation phase), the difficulty level was either fixed, which is termed “fixed-rank” paradigm, or kept adaptive, in the “adaptive-rank” paradigm. In both cases, testing was assumed to be conducted for a period of up to 4 years with weekly testing, i.e., for a total of 220 runs.

The cutoffs for rank change were determined in simulations performed in the context of the design of a clinical study, which is currently in the active recruitment phase. There, the aim was to find a set of cutoffs that enable a rather broad spread in the ranks at study end (36 runs), with the objective of reflecting the high variability in cognitive ability expected in study participants. Parameters of the patient and the study model can be found in the Supplementary material (Supplementary Table S2).

#### 2.2.3 Parameters of the study simulations

Sensitivity to between-group effects, reflective of potential pharmacodynamic responsiveness of candidate biomarkers of Alzheimer’s disease modification, was assessed in five cohorts characterized by cognitive decline rates of zero/run (0%/year), 0.0005/run (~2.5%/year), 0.001/run (~5%/year), 0.0015/run (~7.5%/year) and 0.002/run (~10%/year). As a rough reference, normal aging is expected to be associated with a rate of cognitive performance decline of 0–2.5%/year and appears to be rather linearly stable over the age span, as recently assessed in the INTUITION Study (Butler et al., 2025). Transition from Mild Cognitive Impairment (MCI) to the earliest stages of mild dementia as seen in early AD can be approximately associated with rates of decline evolving between 2.5 and 5% per year (van der Veere et al., 2023). The two higher rates (7.5%/year and 10%/year) correspond to examples anchored in the range of cognitive decline expected in rapidly progressive dementias associated with Alzheimer’s disease (van der Veere et al., 2023) and related disorders.

We simulated cohorts of 200 patients, roughly reflecting a typical small-size phase 2a proof-of-concept study of a new drug or of an observational study investigating cohorts defined by different risk profiles for cognitive decline, for instance subjects at risk for Alzheimer’s disease or with MCI aged 50 years or older.

#### 2.2.4 Endpoints

The rank and score of the last run of the run-in phase were used as baseline for the subsequent evaluation phase. The numerical variables tracked in the evaluation phase to derive longitudinal endpoints were the score for the fixed-rank and the rank for the adaptive-rank paradigm. The reason for this difference is that tracking the score in the adaptive-rank configuration would not provide a consistent and interpretable endpoint: when the rank changes, the score follows a different distribution and has a different longitudinal correlation pattern.

From the longitudinal values, three endpoints were derived for analysis purposes: the change from baseline, the percentage change from baseline and the “time to confirmed decline.” For the latter endpoint, a decline event was defined as a >30% decrease from baseline, and for the event to qualify for the analysis (“confirmed decline”) that level of decline was required to be maintained for a confirmation period of at least eight consecutive runs (reflecting about 2 months).

#### 2.2.5 Analysis methods

The analysis was exploratory and primarily used descriptive statistical methods. Time-to-event endpoints were analyzed using the Kaplan–Meier method. For continuous endpoints, Cohen’s *d* was used as a measure of the effect size when comparing cohorts with different cognitive decline rates.

## 3 Results

We exemplify the key observations using one of CoGames’ ACAs (Numbers), noting that findings were consistent for the other ACA instruments of the CoGames battery (Supplementary Figure S3).

**Figure 2:**
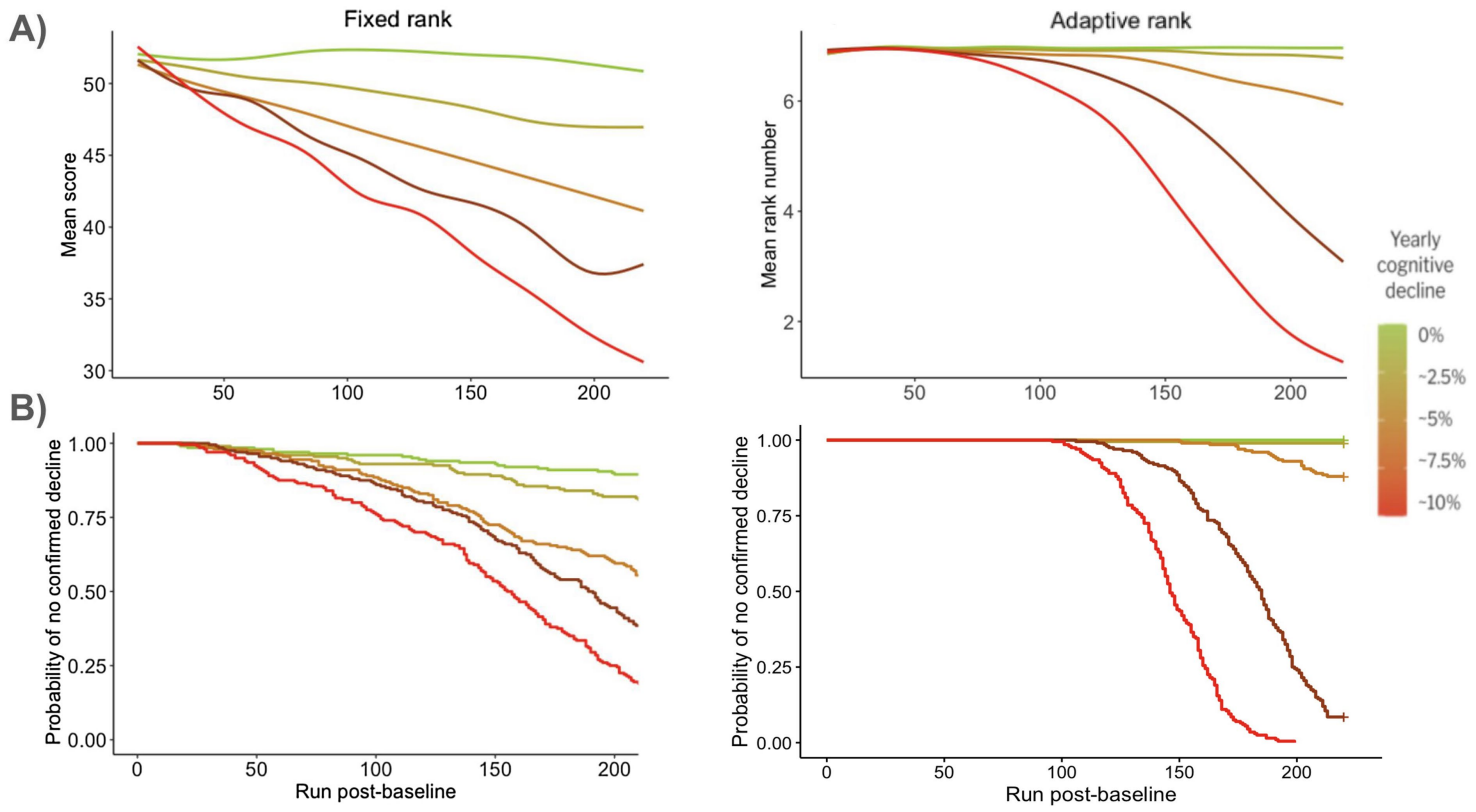
Performance for one ACA instrument (Numbers) in a 4-year study. Comparison of post-baseline fixed difficulty (“Fixed rank”) vs. post-baseline dynamic adjustment of difficulty (“Adaptive rank”). **(A)** Mean values over time; **(B)** Kaplan–Meier analysis of the time to confirmed decline.

Sensitivity to change and between-groups discriminative ability (reflecting pharmacodynamic responsiveness of candidate biomarkers of disease modification) of the Fixed vs. Adaptive rank testing paradigms differed according to parameters of the cohorts/studies. Figure 2 depicts the trajectory of the mean score/rank values over time (Figure 2A) and the analysis of the time to confirmed decline (Figure 2B) for five discrete rates of cognitive decline between 0 and 10%/year. In the fixed-rank paradigm, for both types of analysis, the separation of the curves is rather gradual both as a function of time (i.e., run number) and of the decline rate. In contrast, for the adaptive-rank paradigm, the separation appears non-linear both as a function of time and decline rate: i.e., for the first 1–2 years there is no or minimal separation, but then trajectories separate quickly in particular for the intermediate range of the evaluated rates of decline, corresponding to 2.5–7.5% per year.

**Figure 3:**
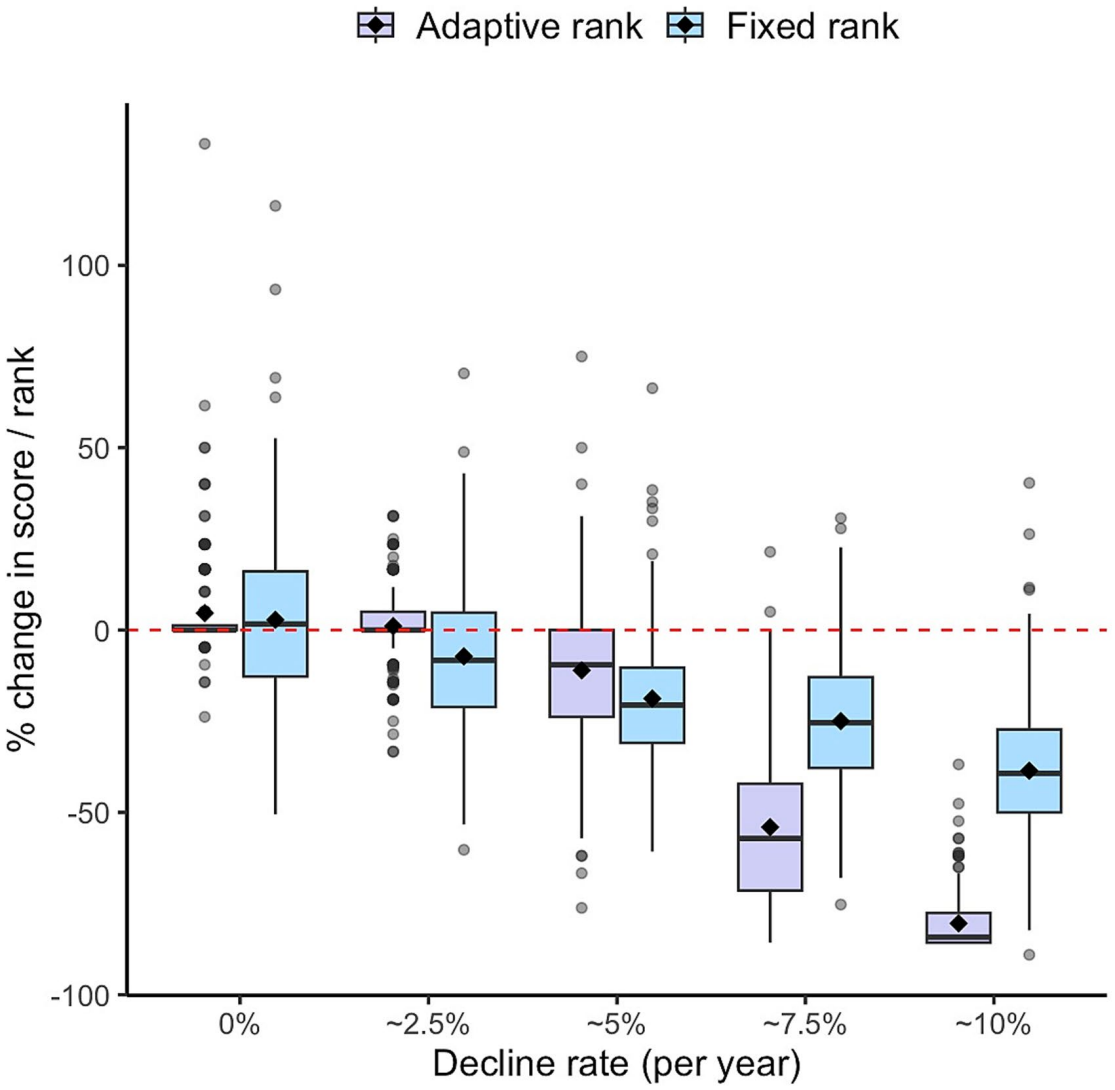
Sensitivity to change of one of the ACA instruments (Numbers) measured by the percentage change in score or rank at end of a 4-year study simulation for the fixed versus adaptive rank paradigms by yearly cognitive decline rate.

Figure 3 visualizes the sensitivity to change, in a 4-year study simulation of one of the ACA instruments (Numbers), as measured by the percentage change in score or rank years for the fixed versus adaptive rank paradigms by yearly cognitive decline rate. At 4 years, the adaptive rank paradigm clearly outperforms the fixed-rank paradigm in terms of sensitivity to change for cognitive decline rates >2.5% per year.

The adaptive rank paradigm outperformed the fixed rank paradigm also in terms of ability to discriminate, e.g., between decline rates of 5 and 7.5%, as measured by Cohen’s *d* calculated for different types of endpoints (Table 1).

**Table 1:** Responsiveness assessed by the effect size at end of a 4-year study simulation for the ability to discriminate between decline rates of 5 and 7.5% with the fixed versus adaptive rank paradigms.

Post-baseline ACA configuration	Effect size type	Study end value	Endpoint change from baseline	%-change from baseline
Fixed difficulty/rank	Raw Cohen’s *d*	0.58	0.32	0.37
Fixed difficulty/rank	Cohen’s *d* from linear model	0.59	0.42	0.45
Adaptive difficulty/rank	Raw Cohen’s *d*	2.09	2.07	2.05
Adaptive difficulty/rank	Cohen’s *d* from linear model	2.10	1.99	1.97

Cohen’s *d* calculated for different types of endpoints and for raw data or based on a linear model with adjustment for baseline.

The theoretical expected effect size of the ACA instrument (Numbers) in the fixed-rank configuration to compare two decline rates separated by 2.5% per year is 0.62 for the most common rank at baseline in the simulations, which is close to the value obtained from simulations. For other baseline ranks, the theoretical effect size goes up to 1.03, which remains clearly smaller than the effect in the adaptive-rank configuration for the comparison of decline rates of 5 and 7.5%.

Because of the described non-linear relationships, there are regions of the parameter space where the sensitivity to treatment effect for the adaptive-rank paradigm is small or even absent.

For example, for the smallest overall decline rates (<5%), the fixed-rank paradigm delivers roughly stable Cohen’s *d* values for the same absolute difference, but the advantage of the adaptive-rank paradigm would decrease: it would still be clear for 2.5% vs. 5% (Cohen’s *d* of 0.95 for the study end values and only slightly lower for the other continuous endpoints), but practically absent for the comparison of 0% vs. 2.5% (Cohen’s *d*: 0.52–0.29).

As illustrated in Figure 2, sensitivity to change and pharmacodynamic responsiveness are dependent on study duration, and findings observed at 4 years would not necessarily generalize to much shorter studies. As a matter of fact, for a readout at 2 years post-baseline, the analysis of the time to confirmed decline with the adaptive-rank paradigm would not be promising because there would be hardly any progression events. For the continuous endpoints, the results in 2-year study simulations are somewhat opposite to 4-year simulations, with a numerical superiority of the fixed-rank paradigm in terms of sensitivity to change and pharmacodynamic responsiveness, albeit with expectedly lower effect sizes (Supplementary Figure S2 and Supplementary Table S3). However, different choices of the ACA parameter may create a shift in these effects.

## 4 Discussion

With the progress in the biological understanding of neurodegenerative diseases such as Alzheimer’s and the identification of druggable targets, the number of interventional trials targeting cognitive decline is increasing (Lysandropoulos, 2024). The success of these trials crucially depends on the sensitivity to detect treatment effects of their cognitive endpoints. In this paper, we investigated Indivi’s battery of novel and gamified adaptive cognitive assessments (ACAs) that are expected to be superior to currently available instruments because the difficulty of the task can be tailored to the capacity of each individual. The aspirational context of use of these instruments in clinical development programs may be that of pharmacodynamic response biomarkers indicative of disease modification (Cummings et al., 2025).

Adaptive tests produce complex longitudinal trajectories, which experimental data and statistical models from a limited set of study configurations can only capture to a limited extent. This motivated us to develop a simulation tool for longitudinal studies of subjects performing our adaptive cognitive assessments. The description of the modeling approach is a first contribution of our work to the literature.

In simulations targeting cohorts of subjects with, or at risk of, rapid cognitive decline, we specifically investigated two deployment paradigms: with or without rank adaptation after an initial adaptive pre-baseline run-in phase, which was meant to saturate learning/practice effects. We found that the sensitivity to treatment effects was robustly different for the two paradigms. The enhanced sensitivity in the adaptive rank paradigm crucially depends on the presence of dynamic features in the cognitive assessments, which confer a means of amplifying treatment effects in specific regions of the parameter space.

Several limitations have to be considered. First, the simulations may not capture relevant aspects of the cognitive trajectories, and refinements of the patient models are planned based on data currently being collected at different stages of the Alzheimer’s disease continuum, including pre-symptomatic subjects. Preliminary analyses suggest that the models are reasonable, although much larger datasets would be needed for a robust assessment. Second, although our findings are robust and concern practically relevant scenarios, we cannot claim any generality, neither for the parameter range we explored, nor for the resulting interpretation. In this sense, our findings have to be considered an illustration of specific emergent properties of the system rather than general rules that practitioners can directly apply to their study. Any concrete study design will require a careful evaluation of a large parameter space. Some parameters will be constrained by features of the study (e.g., the characteristics of the subjects in a specific therapeutic indication), but there are many parameters that the experimenter will be able to (almost) freely manipulate and that have a large influence on the measurement properties of the system. In addition to the discussed endpoint configuration and study duration, important free parameters in the study model are the cutoffs for changing rank, which strongly impact the adaptive rank configuration.

Simulations are becoming a standard tool at the planning stage of clinical studies, including for investigations using digital measures in neurodegenerative diseases (Lavine et al., 2024). We found simulations to be useful for the concrete task of determining these cutoffs for non-comparative studies in already two therapeutic areas, and they will be indispensable for the evaluation of comparative study designs.

In conclusion, we developed a tool to simulate clinical studies using a battery of novel gamified ACAs. Simulations showed that with ACAs, post-baseline difficulty adaptation can provide increased sensitivity to cognitive decline and to treatment effects over fixed difficulty in longer study durations. Customized use of ACAs should be guided by study design features, such as duration, ACA administration frequency, expected levels of impairment, decline and treatment effect in target populations.

## Data Availability

The raw data supporting the conclusions of this article will be made available by the authors without undue reservation.

## References

[ref1] Aarsland D. Creese B. Politis M. Chaudhuri K. R. Ffytche D. H. Weintraub D.. (2017). Cognitive decline in Parkinson disease. Nat. Rev. Neurol. 13, 217–231. doi: 10.1038/nrneurol.2017.27, PMID: 28257128 PMC5643027

[ref2] Albert M. S. DeKosky S. Dickson D. Dubois B. Feldman H. H. Fox N. C.. (2011). The diagnosis of mild cognitive impairment due to Alzheimer’s disease: recommendations from the National Institute on Aging-Alzheimer’s association workgroups on diagnostic guidelines for Alzheimer’s disease. Alzheimers Dement. 7, 270–279. doi: 10.1016/j.jalz.2011.03.008, PMID: 21514249 PMC3312027

[ref3] Bartels C. Wegrzyn M. Wiedl A. Ackermann V. Ehrenreich H. (2010). Practice effects in healthy adults: a longitudinal study on frequent repetitive cognitive testing. BMC Neurosci. 11:118. doi: 10.1186/1471-2202-11-118, PMID: 20846444 PMC2955045

[ref4] Benedict R. H. Kappos L. Miller A. Hartung H. P. Overell J. Pei J.. (2025). Cognitive effects of ocrelizumab vs interferon β-1a in relapsing multiple sclerosis: a post hoc analysis of the OPERA I/II trials. Mult. Scler. Relat. Disord. 95:106310. doi: 10.1016/j.msard.2025.106310, PMID: 39965438

[ref5] Butler P. M. Yang J. Brown R. Hobbs M. Becker A. Penalver-Andres J.. (2025). Smartwatch- and smartphone-based remote assessment of brain health and detection of mild cognitive impairment. Nat. Med. 31, 829–839. doi: 10.1038/s41591-024-03475-9, PMID: 40038507 PMC11922773

[ref6] Cummings J. L. Teunissen C. E. Fiske B. K. le Ber I. Wildsmith K. R. Schöll M.. (2025). Biomarker-guided decision making in clinical drug development for neurodegenerative disorders. Nat. Rev. Drug Discov. 24, 589–609. doi: 10.1038/s41573-025-01165-w, PMID: 40185982

[ref7] Elkana O. Eisikovits O. R. Oren N. Betzale V. Giladi N. Ash E. L. (2016). Sensitivity of neuropsychological tests to identify cognitive decline in highly educated elderly individuals: 12 months follow up. J. Alzheimer's Dis. 49, 607–616. doi: 10.3233/JAD-150562, PMID: 26484925

[ref8] Gibbons R. D. Lauderdale D. S. Wilson R. S. Bennett D. A. Arar T. Gallo D. A. (2024). Adaptive measurement of cognitive function based on multidimensional item response theory. Alzheimer’s Dement. Transl. Res. Clin. Interv. 10:e70018. doi: 10.1002/trc2.70018, PMID: 39748843 PMC11694520

[ref9] Lam K.-H. Bucur I. G. van Oirschot P. de Graaf F. Weda H. Strijbis E.. (2022). Towards individualized monitoring of cognition in multiple sclerosis in the digital era: a one-year cohort study. Mult. Scler. Relat. Disord. 60:103692. doi: 10.1016/j.msard.2022.103692, PMID: 35219240

[ref10] Lavine J. S. Scotina A. D. Haney S. Bakker J. P. Izmailova E. S. Omberg L. (2024). Impacts on study design when implementing digital measures in Parkinson’s disease-modifying therapy trials. Front. Digit. Health 6:1430994. doi: 10.3389/fdgth.2024.1430994, PMID: 39445101 PMC11496294

[ref11] Leavitt V. Mostert J. Comtois J. Moral E. Brieva L. Repovic P.. (2025). Measuring cognitive change in secondary progressive MS: an analysis of the ASCEND cognition substudy. J. Neurol. 272:338. doi: 10.1007/s00415-025-13066-4, PMID: 40220155

[ref12] Lysandropoulos A. (2024). Current trends in neuroscience clinical research and regulatory landscape (P7-4.008). Neurology 102:3288. doi: 10.1212/WNL.0000000000205040

[ref13] Mintun M. A. Lo A. C. Duggan Evans C. Wessels A. M. Ardayfio P. A. Andersen S. W.. (2021). Donanemab in early Alzheimer’s disease. N. Engl. J. Med. 384, 1691–1704. doi: 10.1056/NEJMoa2100708, PMID: 33720637

[ref14] Nasirzadeh A. Mohammadi M. Bafrani M. A. Mohammadi A. Bakhtiari-Dovvombaygi H. (2024). Comparing cognitive impairment using MACFIMS in patients with multiple sclerosis and healthy controls: a systematic review and meta-analysis. BMC Neurol. 24:454. doi: 10.1186/s12883-024-03943-2, PMID: 39563246 PMC11575216

[ref15] Paulsen J. S. Smith M. M. Long J. D. the PREDICT HD investigators and coordinators of the Huntington Study Group (2013). Cognitive decline in prodromal Huntington disease: implications for clinical trials. J. Neurol. Neurosurg. Psychiatry 84, 1233–1239. doi: 10.1136/jnnp-2013-305114, PMID: 23911948 PMC3795884

[ref16] Pham L. Harris T. Varosanec M. Morgan V. Kosa P. Bielekova B. (2021). Smartphone-based symbol-digit modalities test reliably captures brain damage in multiple sclerosis. NPJ Digit. Med. 4:36. doi: 10.1038/s41746-021-00401-y, PMID: 33627777 PMC7904910

[ref17] Pless S. Woelfle T. Lorscheider J. Wiencierz A. Reyes Ó. Luque C.. (2025). CoGames: development of an adaptive smartphone-based and gamified monitoring tool for cognitive function in multiple sclerosis. J. Neurol. 272:119. doi: 10.1007/s00415-024-12818-y, PMID: 39812703 PMC11735570

[ref18] Pless S. Woelfle T. Naegelin Y. Lorscheider J. Wiencierz A. Reyes Ó.. (2023). Assessment of cognitive performance in multiple sclerosis using smartphone-based training games: a feasibility study. J. Neurol. 270, 3451–3463. doi: 10.1007/s00415-023-11671-9, PMID: 36952010 PMC10267276

[ref19] R Core Team (2021). R: a language and environment for statistical computing. Vienna, Austria: R Foundation for Statistical Computing.

[ref20] Research Center for Clinical Neuroimmunology and Neuroscience Basel. (2025). DreaMS-development of digital biomarkers in multiple sclerosis-validation study 1. Clinical trial registration NCT05009160. clinicaltrials.gov. Available online at: https://clinicaltrials.gov/study/NCT05009160 (Accessed June 19, 2025).

[ref21] Strober L. DeLuca J. Benedict R. H. B. Jacobs A. Cohen J. A. Chiaravalloti N.. (2019). Symbol digit modalities test: a valid clinical trial endpoint for measuring cognition in multiple sclerosis. Mult. Scler. J. 25, 1781–1790. doi: 10.1177/1352458518808204, PMID: 30334474 PMC6826875

[ref22] van der Veere P. J. Hoogland J. Visser L. N. C. van Harten A. C. Meester H. F. M. R. Barkhof F.. (2023). Predicting cognitive decline in amyloid-positive patients with mild cognitive impairment or mild dementia. Alzheimers Dement. 19:e082589. doi: 10.1002/alz.082589, PMID: 38986053 PMC11238942

[ref23] van Dyck C. H. Swanson C. J. Aisen P. Bateman R. J. Chen C. Gee M.. (2023). Lecanemab in early Alzheimer’s disease. N. Engl. J. Med. 388, 9–21. doi: 10.1056/NEJMoa2212948, PMID: 36449413

[ref24] Wouters H. Zwinderman A. H. van Gool W. A. Schmand B. Lindeboom R. (2009). Adaptive cognitive testing in dementia. Int. J. Methods Psychiatr. Res. 18, 118–127. doi: 10.1002/mpr.283, PMID: 19507163 PMC6878369

[ref25] Zheng B. Udeh-Momoh C. Watermeyer T. de Jager Loots C. A. Ford J. K. Robb C. E.. (2022). Practice effect of repeated cognitive tests among older adults: associations with brain amyloid pathology and other influencing factors. Front. Aging Neurosci. 14:909614. doi: 10.3389/fnagi.2022.909614, PMID: 35875808 PMC9297730

