# Supplementary material for: Biomarkers of Alzheimer’s disease modification using adaptive cognitive assessments to improve responsiveness—a simulation study
Source: Front Neurosci. 2025 Sep 23;19:1653261. doi: 10.3389/fnins.2025.1653261 (PMC12500584; doi:10.3389/fnins.2025.1653261)
Supplement: Supplementary file 1 [file Data_Sheet_1.docx]

Supplementary Material

# Supplementary Data

**Figure S1: CoGames ACA battery of instruments**Adapted from: Pless, S. et al. Journal of Neurology (2025), 272(2), p. 119 - doi.org/10.1007/s00415-024-12818-y.


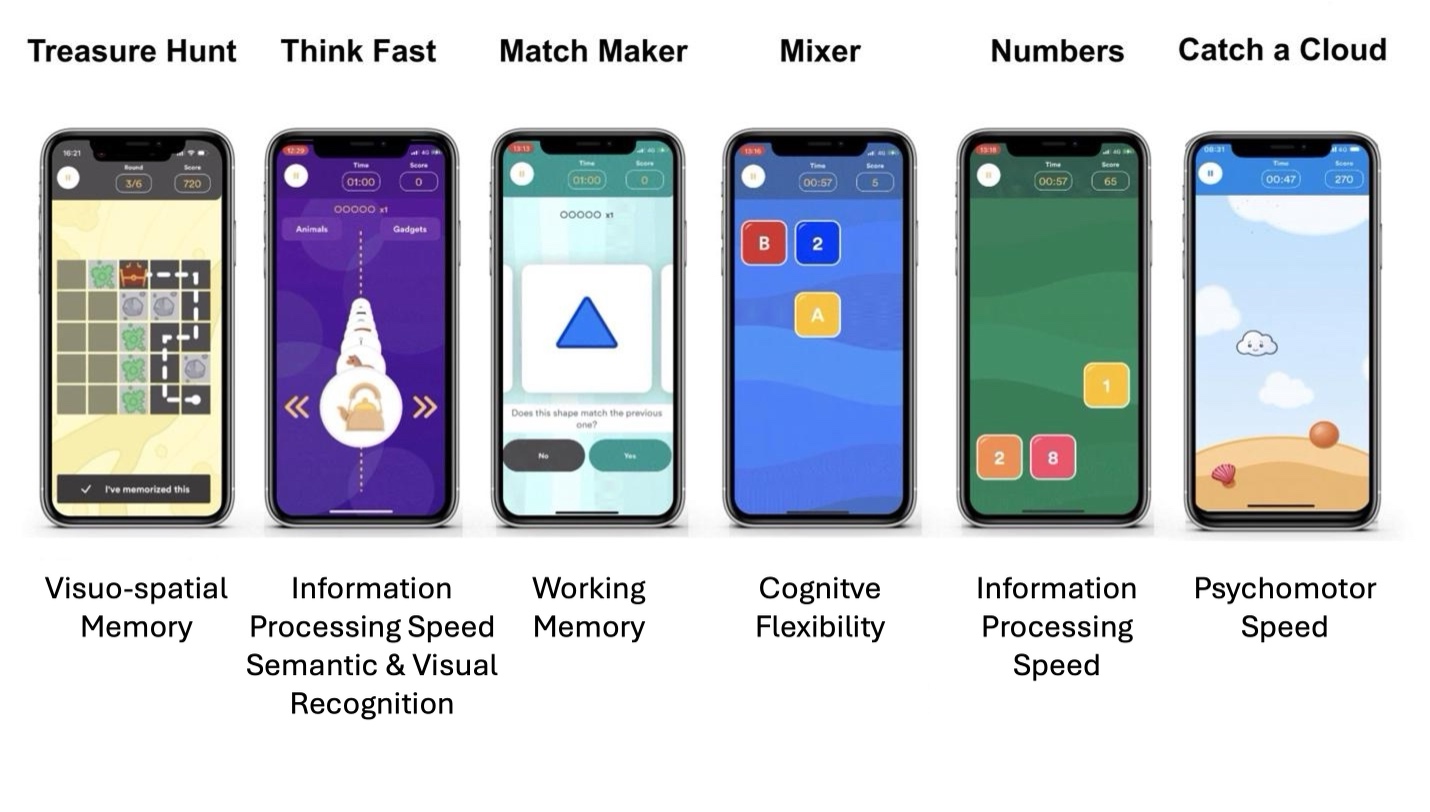


**Table S1:** **Cognitive domains, ACA instrument, measures, description**
Adapted from Pless *et al.*, 2025

| **Main Cognitive**  **Domain** | **ACA**  **instrument**  (No. of Ranks) | **Primary measure** | **Description** |
| --- | --- | --- | --- |
| Working memory | Match Maker  (4 ranks) | Number of correct responses in 60 sec. | The system shows an either colorful or grey shape on the screen. With every answer, a new shape appears and the previous is hidden. The task is to continuously decide (“Yes”/”No”) whether the presented shape matches the one shown “n” taps before. “N” increases with higher difficulty. |
| Information processing speed | Think Fast  (7 ranks) | Number of correct responses in 60 sec. | The system shows an image which must be sorted into the correct category as fast as possible. The number of categories and their similarity change with difficulty. |
|  | Numbers  (7 ranks) | Number of correct responses in 60 sec. | The system displays a series of numbers that are randomly placed across the screen. The participant’s task is to tap the numbers in ascending order, as fast as possible. |
| Visuo-spatial memory | Treasure Hunt  (8 ranks) | Mean percentage of correct grid-square connections over 6 rounds | The system shows a grid with a path to an “X” (treasure). The participant must memorize the exact path to the treasure. After an intermission time the participant must reconstruct the memorized path on a blank grid. Grid size, path length and intermission time length are increased with higher difficulty. |
| Cognitive flexibility | Mixer  (5 ranks) | Number of correct responses in 60 sec. | The system displays a series of letters and numbers that are randomly placed across the screen. The participant must tap the numbers and letters in ascending-/ alphabetical order, as fast as possible, always alternating between the two. |
| Psychomotor speed | Catch a Cloud  (6 ranks) | Number of correct responses in 30 sec. | The system displays clouds on screen which must be “tapped” as fast as possible. Once tapped, the cloud reappears at another location on the screen. The first 20 seconds (*Practice Mode*) only shows 1 cloud on screen. After the *Practice Mode*, the *Game Mode* follows for 30 seconds. Higher levels of the *Game Mode* include more gamification elements such as raining clouds, distractions (Balloons) which must be avoided, and multiple clouds at once. |

**Table S2: Parameters of the Patient and the Study Model**n1; n2: number of subjects used for the derivation of the parameters for the 1^st^ and 2^nd^ repetition

| **ACA**  **instrument** | **Rank** |  | **Patient Model** (distribution parameters - not accounting for learning and decline) | | | | **Study Model**  (rank transition cutoffs) | |
| --- | --- | --- | --- | --- | --- | --- | --- | --- |
|  |  | **n1; n2** | **SD 1st repetition** | **SD 2nd repetition** | **Mean 1st repetition** | **Mean 2nd repetition** | **Score cutoff for ranking up** | **Score cutoff for ranking down** |
| Numbers | 1 | 187; 177 | 7.349 | 6.779 | 51.246 | 51.734 | 44 | NA |
| Numbers | 2 | 147; 127 | 8.813 | 8.279 | 47.31 | 49.346 | 48 | 39 |
| Numbers | 3 | 126; 122 | 8.58 | 7.745 | 48.659 | 48.959 | 52 | 43 |
| Numbers | 4 | 71; 67 | 8.446 | 9.73 | 46.52 | 46.119 | 50 | 41 |
| Numbers | 5 | 75; 72 | 8.706 | 8.708 | 42.201 | 43.101 | 48 | 37 |
| Numbers | 6 | 69; 67 | 8.293 | 8.274 | 42.002 | 41.851 | 51 | 39 |
| Numbers | 7 | 69; 66 | 8.783 | 8.586 | 39.007 | 39.346 | NA | 36 |
| Mixer | 1 | 186; 177 | 9.305 | 9.164 | 46.694 | 50.695 | 39 | NA |
| Mixer | 2 | 137; 126 | 7.849 | 7.688 | 47.891 | 49.381 | 51 | 43 |
| Mixer | 3 | 71; 67 | 7.863 | 8.963 | 39.925 | 41.926 | 43 | 35 |
| Mixer | 4 | 75; 72 | 9.459 | 8.769 | 39.272 | 41.843 | 48 | 35 |
| Mixer | 5 | 69; 67 | 9.667 | 8.041 | 40.638 | 41.377 | NA | 38 |
| Think Fast | 1 | 188; 177 | 19.449 | 20.967 | 64.601 | 72.034 | 43 | NA |
| Think Fast | 2 | 134; 120 | 17.531 | 19.156 | 71.149 | 76.042 | 72 | 54 |
| Think Fast | 3 | 118; 105 | 17.153 | 15.99 | 77.331 | 80.248 | 83 | 66 |
| Think Fast | 4 | 116; 101 | 17.257 | 17.798 | 64.078 | 66.257 | 70 | 53 |
| Think Fast | 5 | 76; 73 | 15.671 | 14.137 | 68.993 | 70.771 | 75 | 59 |
| Think Fast | 6 | 75; 74 | 16.048 | 16.737 | 59.385 | 64.997 | 71 | 49 |
| Think Fast | 7 | 71; 66 | 14.902 | 13.242 | 57.596 | 63.039 | NA | 51 |
| Match Maker | 1 | 186; 176 | 11.415 | 12.836 | 53.935 | 57.295 | 47 | NA |
| Match Maker | 2 | 71; 66 | 11.889 | 11.62 | 61.283 | 61.366 | 66 | 54 |
| Match Maker | 3 | 69; 66 | 10.152 | 9.953 | 43.477 | 42.691 | 51 | 40 |
| Match Maker | 4 | 69; 66 | 7.775 | 7.411 | 34.288 | 34.751 | NA | 32 |
| Treasure Hunt | 1 | 186; 175 | 6.608 | 6.193 | 95.426 | 95.645 | 88 | NA |
| Treasure Hunt | 2 | 152; 131 | 9.63 | 8.878 | 91.099 | 91.413 | 92 | 82 |
| Treasure Hunt | 3 | 141; 125 | 11.319 | 11.924 | 87.111 | 88.477 | 88 | 76 |
| Treasure Hunt | 4 | 90; 81 | 9.683 | 11.589 | 87.179 | 86.336 | 91 | 81 |
| Treasure Hunt | 5 | 75; 71 | 18.062 | 17.55 | 73.706 | 75.235 | 80 | 62 |
| Treasure Hunt | 6 | 75; 70 | 21.12 | 21.969 | 71.957 | 70.223 | 87 | 58 |
| Treasure Hunt | 7 | 69; 65 | 22.95 | 24.841 | 69.667 | 69.6 | 97 | 61 |
| Treasure Hunt | 8 | 69; 65 | 22.695 | 23.028 | 70.341 | 71.821 | NA | 62 |
| Catch a Cloud | 1 | 192; 186 | 7.903 | 7.572 | 40.229 | 41.634 | 33 | NA |
| Catch a Cloud | 2 | 122; 107 | 5.941 | 6.307 | 46.516 | 46.486 | 47 | 41 |
| Catch a Cloud | 3 | 96; 81 | 5.89 | 6.474 | 50.698 | 51.383 | 51 | 45 |
| Catch a Cloud | 4 | 68; 56 | 5.736 | 5.854 | 47.574 | 47.143 | 51 | 44 |
| Catch a Cloud | 5 | 53; 41 | 4.954 | 5.255 | 50.811 | 51.293 | 56 | 48 |
| Catch a Cloud | 6 | 59; 53 | 7.091 | 7.011 | 50.695 | 50.642 | NA | 48 |

**Figure S2: Sensitivity to change of one of the ACA instruments.** Display of the percentage change in score or rank at end of a 2-year study simulation using the “Numbers” instrument, for the fixed versus adaptive rank paradigms, by yearly cognitive decline rate.

**
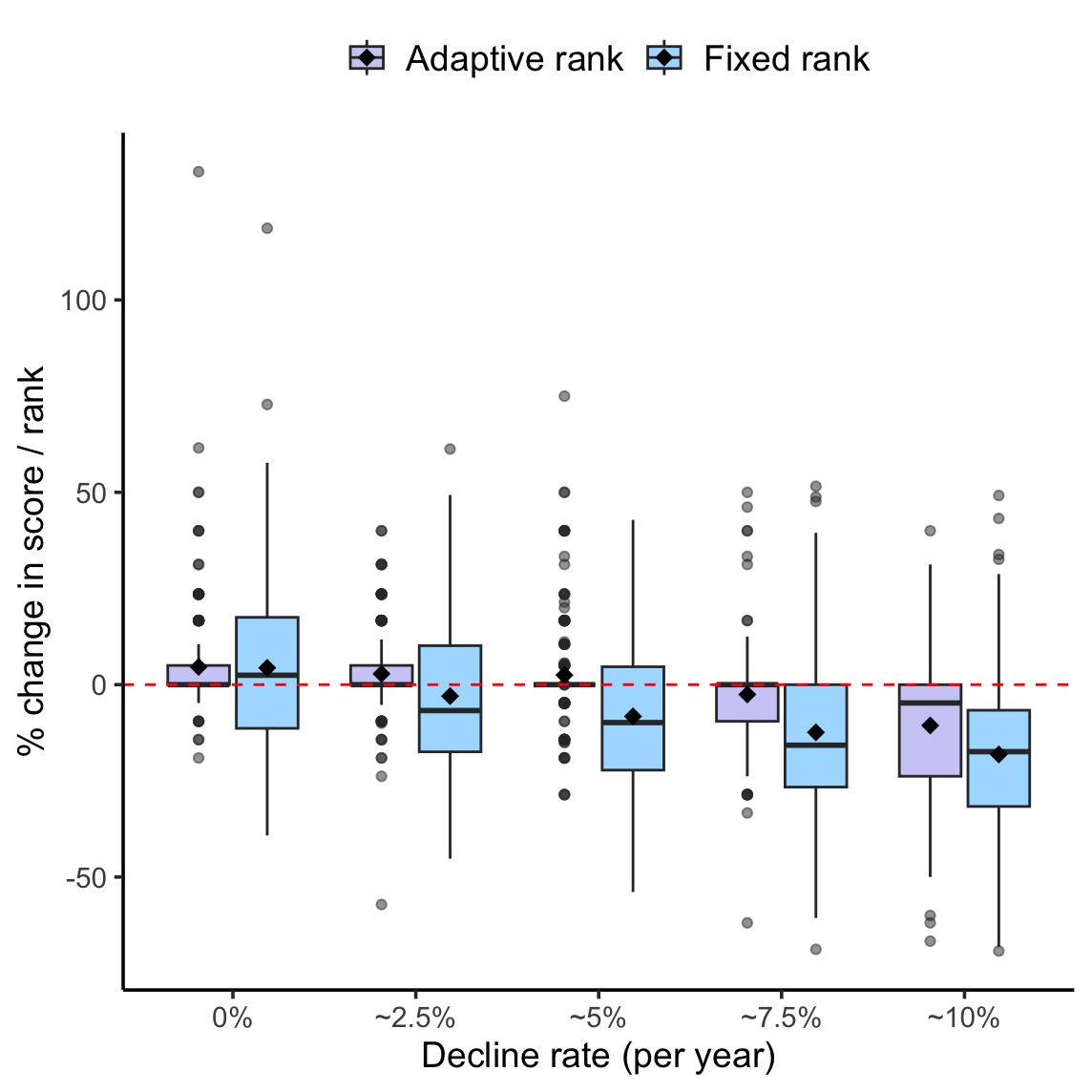
**

**Table S3: Responsiveness.**  Effect size at end of a 2-year study simulation for the ability to discriminate between decline rates of 5% and 7.5% with the fixed versus adaptive rank paradigms. Cohen’s d calculated for different types of endpoints and for raw data or based on a linear model with adjustment for baseline.

| **Post-baseline ACA Configuration** | **Effect size type** | **Study end value** | **Endpoints  Change from baseline** | **%-change from baseline** |
| --- | --- | --- | --- | --- |
| Fixed Difficulty/Rank | Raw Cohen’s d | 0.31 | 0.26 | 0.26 |
| Fixed Difficulty/Rank | Cohen’s d from linear model | 0.31 | 0.23 | 0.23 |
| Adaptive Difficulty/Rank | Raw Cohen’s d | 0.15 | 0.07 | 0.03 |
| Adaptive Difficulty/Rank | Cohen’s d from linear model | 0.15 | 0.09 | 0.05 |

**Figure S3: Performance for ACA instrument in a 4-year study**. Comparison of the mean score (left) and rank (right) over time for post-baseline fixed difficulty (“Fixed rank”) vs. post-baseline dynamic adjustment of difficulty (“Adaptive rank”).

**
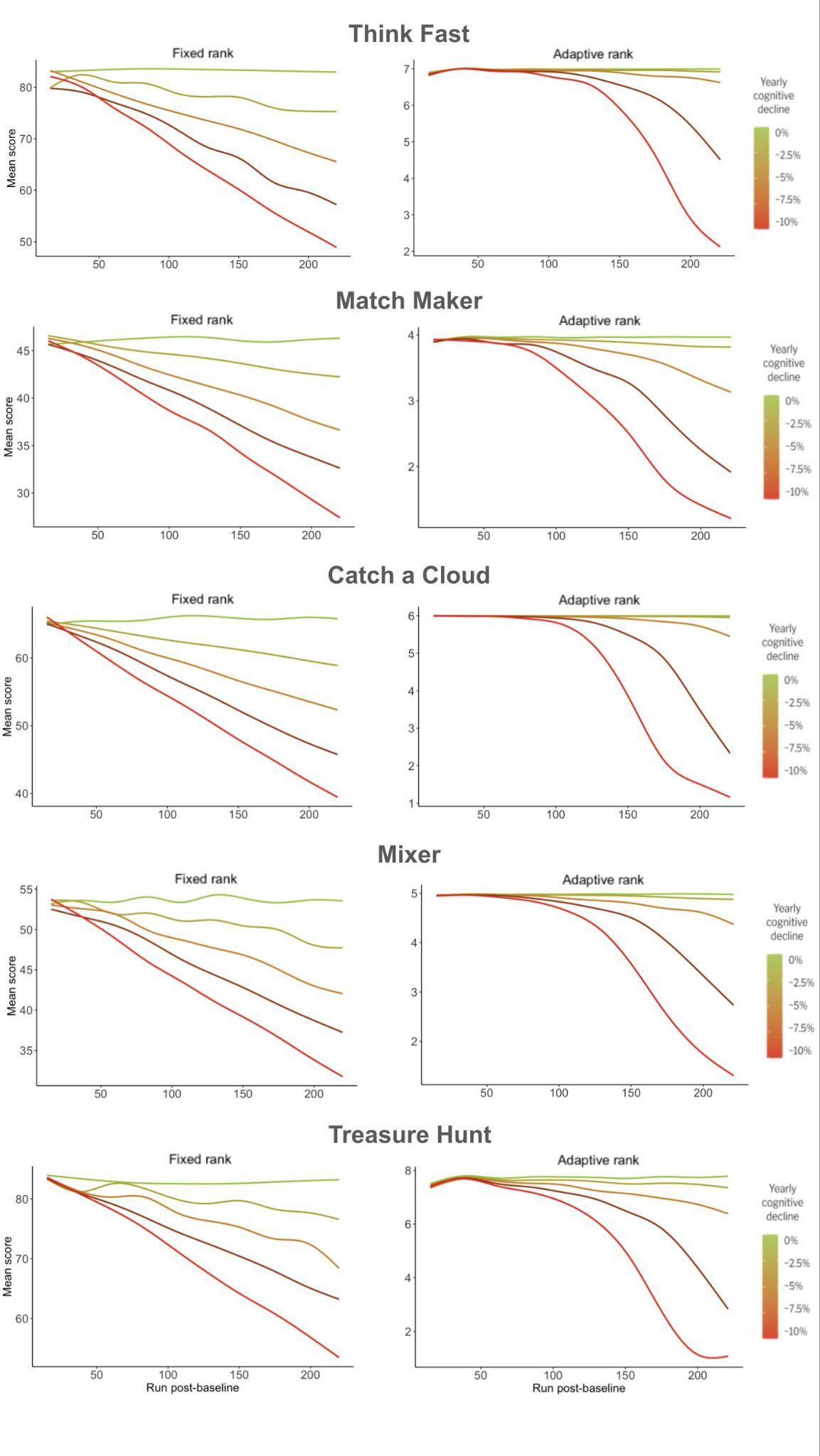
**
